# Optimization of Conditions for In Vitro Culture of the Microphallid Digenean *Gynaecotyla adunca*

**DOI:** 10.1155/2014/382153

**Published:** 2014-03-25

**Authors:** Jenna West, Alexandra Mitchell, Oscar J. Pung

**Affiliations:** Department of Biology, Georgia Southern University, P.O. Box 8042-1, Statesboro, GA 30458, USA

## Abstract

In vitro cultivation of digeneans would aid the development of effective treatments and studies of the biology of the parasites. The goal of this study was to optimize culture conditions for the trematode, *Gynaecotyla adunca*. Metacercariae of the parasite from fiddler crabs, *Uca pugnax*, excysted in trypsin, were incubated overnight to permit fertilization, and were cultured in different conditions to find those that resulted in maximum worm longevity and egg production. When cultured in media lacking serum, worms lived longer in Hanks balanced salt solution and Dulbecco's Modified Eagle medium/F-12 (DME/F-12) than in RPMI-1640 but produced the most eggs in DME/F-12. Worm longevity and egg production increased when worms were grown in DME/F-12 supplemented with 20% chicken, horse, or newborn calf serum but the greatest number of eggs was deposited in cultures containing horse or chicken serum. Horse serum was chosen over chicken serum due to the formation of a precipitate in chicken serum. The optimal concentration of horse serum with respect to egg production ranged from 5 to 20%. Infectivity of eggs deposited by worms in culture was tested by feeding eggs to mud snails, *Ilyanassa obsoleta*. None of these snails produced *G. adunca* cercariae.

## 1. Introduction

The ability to grow all of the life stages of a digenean in vitro would benefit development of anthelmintic drugs and vaccines [1] and facilitate research into the ecology, evolution, genetics, and behavior of these parasites [2–5]. Though larval stages of some digeneans have been cultured successfully [6–9], most attempts to develop in vitro culture systems that fully replace the definitive host have failed. Excysting metacercariae of other digeneans including* Microphalloides japonicus* [10],* Cotylurus strigeoides* [11],* Microphallus similis* [12],* Haplorchis taichui* [13], and* Maritrema novaezealandensis* [5] can be maintained up to several days in vitro and mature into ovigerous adults, but the eggs produced are frequently malformed or unembryonated (reviewed by [2, 3, 14]). An exception is the culture system developed by Basch et al. [15], who grew the strigeid* Cotylurus lutzi* from metacercariae to adults that produced embryonated eggs yielding miracidia infectious to* Biomphalaria glabrata* snails.

We reported the successful in vitro cultivation of a microphallid digenean,* Microphallus turgidus*, from metacercaria to reproductive adult [16, 17]. Metacercariae of the parasite obtained from the grass shrimp second intermediate host,* Palaemonetes pugio*, mature into egg-depositing adults in our culture system. When the snail first intermediate host,* Spurwinkia salsa*, is fed these eggs in the laboratory, it produces cercariae that are infective to grass shrimp. To our knowledge,* M. turgidus* is only the second digenean and the first microphallid trematode, known to produce viable, infectious eggs in the absence of a definitive host. Because of the uniqueness of this finding, the present study was initiated. The objectives were to (1) optimize the reagents employed in the above protocol to maximize in vitro worm longevity and egg production of another microphallid,* Gynaecotyla adunca*, and to then (2) determine if eggs deposited by the* G. adunca* in vitro were infectious. This is important as it will permit us to determine whether the successful laboratory cultivation of* M. turgidus* was due to an idiosyncrasy of that particular parasite or if the protocol is broadly applicable to other digeneans.

We chose* G. adunca* for this study because, like* M. turgidus*, metacercariae of the parasite are progenetic with well-defined genitalia [18] and should not require long-term culture to develop into egg-producing adults. Also, encysting metacercariae of the parasite are readily available in the green glands of* Uca pugnax* fiddler crabs from the marshes of southeast Georgia. The Eastern mud snail,* Ilyanassa obsoleta*, is the first intermediate host of* G. adunca* [19] and is also abundant. Finally, an early experimental infection study of* G. adunca* [19] suggested that both the parasite and the snail host might be good models for cultivation and infection experiments.

## 2. Materials and Methods

Atlantic mud fiddler crabs,* U. pugnax*, were collected from salt marsh on the Skidaway River in Southeast Georgia (31°56′53.52′′ N, 81°04′13.32′′ W). Crabs were maintained in the laboratory for no longer than 30 days in artificial brackish water (Instant Ocean, Aquarium Systems Inc., Mentor, Ohio) prepared using dechlorinated tap water with salinity adjusted to 23 parts per thousand. Crabs were fed to satiation with tropical fish food flakes (TetraMin, Tetra Werke, Melle, Germany) and given clean water twice a week. Metacercarial cysts of* G. adunca* were removed from fiddler crab green glands and allowed to excyst for 30 min at 42 C in Hanks balanced salt solution (HBSS; HyClone Laboratories, Logan, Utah) containing 0.5% porcine trypsin (HyClone Laboratories, Logan, Utah). HBSS and media used in all studies contained 50 units/mL penicillin and 50 *μ*g/mL streptomycin.

To optimize in vitro egg deposition, excysting worms were first incubated 24 hr at 42 C in 15 mL conical bottom polypropylene centrifuge tubes (50 worms per tube) containing 10 mL HBSS to permit fertilization. An average of 22.6 ± 5.2% of worms were fertilized, that is, had sperm in the seminal receptacle or uterus after this incubation. Worms were then transferred to 48-well polystyrene tissue culture plates (5 worms/well) containing 1 mL/well of HBSS or 1 mL/well of RPMI-1640 or Dulbecco's Modified Eagle medium/F-12 (DME/F-12, both media with HEPES buffer and L-glutamine; HyClone Laboratories, Logan, Utah). In some studies, media were supplemented with horse, newborn calf, or chicken serum (GIBCO, Grand Island, New York). All sera were heat inactivated for 1 hr at 56 C. Worms were cultured at 42 C in a humidified incubator with a gas phase of air and monitored daily for survival. Worms were checked for the presence of eggs in the uterus on Day 3 of culture by visual examination with an inverted microscope (32–100x). After all of the worms had died (longevity studies) or after 10 days of culture, the number of eggs deposited in culture was counted on a hemacytometer.

The infectivity of eggs produced in vitro was determined as follows. Worms were cultured for 10 days as described above in DME/F-12 supplemented with 5% horse serum. Eggs were transferred to artificial brackish water and either immediately fed to Eastern mud snails,* I. obsoleta*, or fed to snails after incubation of eggs at 30 C on a 12 hr light-dark cycle for 10 days. Snails (*n* = 20 in both cases) were exposed to 150 eggs. Snails were then maintained at 30 C on a 12 hr light-dark cycle in artificial brackish water in 1.5 L glass Carolina culture dishes (Carolina Biological Supply Co., Burlington, North Carolina; 25 snails/dish). Snails were fed concentrated microalgae (Shellfish Diet 1800, Instant Algae, Reed Mariculture Inc., Campbell, California) and given fresh water twice a week. Eight weeks after exposure to* G. adunca* eggs, snails were dissected and examined for the presence of* G. adunca* cercariae.

Data were analyzed with JMP 10.0 software. One-way analysis of variance (ANOVA) was used to compare the effects of different media, sera, and serum concentrations on the number of eggs deposited by worms in culture and on worm longevity. The Tukey-Kramer honest significant difference test was used to identify pairs of means that were significantly different. In some cases data did not have a normal distribution and were analyzed as mentioned above following logarithmic transformation. *G*-tests were used to compare the percentages of worms with eggs in utero on Day 3 of culture. Each experiment was performed at least twice and yielded comparable results each time. Analyses and figures represent data from the last trial performed. All data represent the mean values ± 1 SE.

## 3. Results

**Figure 1:**
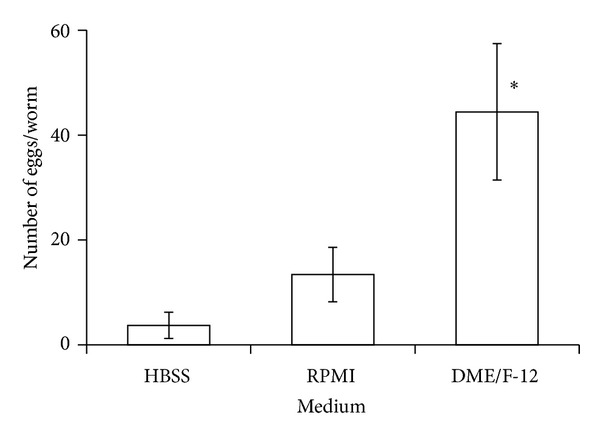
In vitro egg deposition by the trematode* Gynaecotyla adunca* in Hanks balanced salt solution (HBSS) and 2 culture media maintained at 42 C in a gas phase of air. Asterisk indicates significantly different from worms cultured in HBSS. Each value is a mean ± 1 SE.

**Figure 2:**
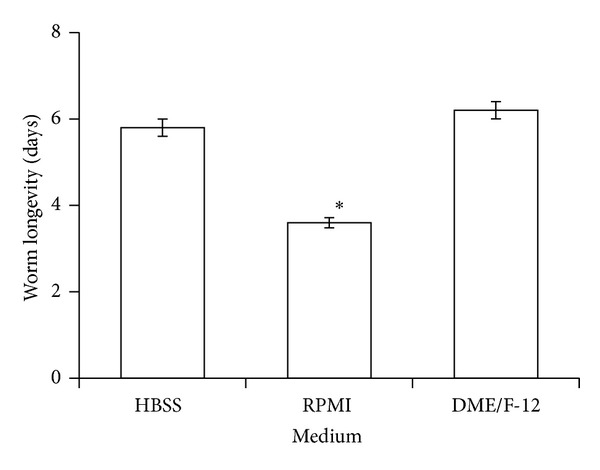
In vitro longevity of the trematode* Gynaecotyla adunca* in Hanks balanced salt solution (HBSS) and 2 culture media maintained at 42 C in a gas phase of air. Asterisk indicates significantly different from worms cultured in HBSS. Each value is a mean ± 1 SE.

There was a significant difference between worms cultured in serum-free HBSS, RPMI-1640, and DME/F-12 with respect to both the number of eggs deposited (ANOVA, df = 2, 21; *F* = 6.5, *P* = 0.006) and worm longevity (ANOVA, df = 2, 122; *F* = 56.8, *P* < 0.0001) but not the percentages of worms producing eggs in utero. Worms deposited the most eggs when cultured in DME/F-12 (Figure 1) and lived longer in HBSS and DME/F-12 than in RPMI 1640 (Figure 2).

**Figure 3:**
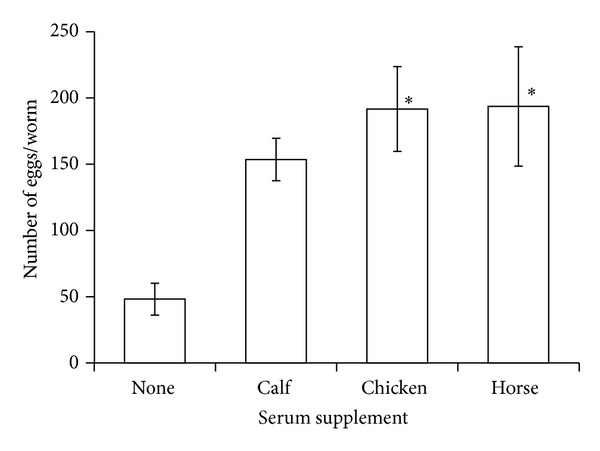
In vitro egg deposition by the trematode* Gynaecotyla adunca* in DME/F-12 with and without 20% animal serum and maintained at 42 C in a gas phase of air. Asterisk indicates significantly different from worms cultured in serum-free DME/F-12 but not from each other. Each value is a mean ± 1 SE.

**Figure 4:**
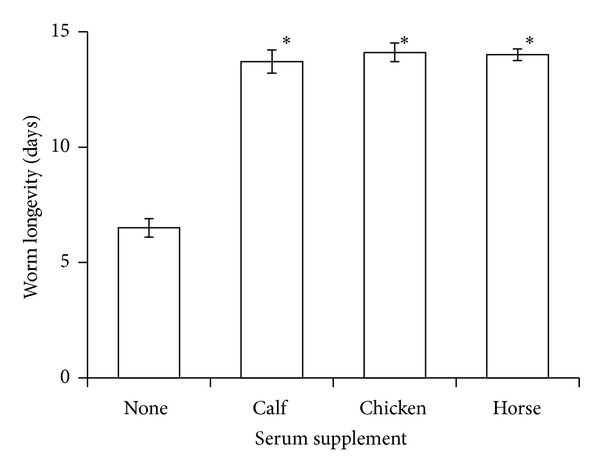
In vitro longevity of the trematode* Gynaecotyla adunca* in DME/F-12 with and without 20% animal serum and maintained at 42 C in a gas phase of air. Asterisk indicates significantly different from worms cultured in serum-free DME/F-12 but not from each other. Each value is a mean ± 1 SE.

The addition of 20% serum to DME/F-12 had a significant effect on egg deposition (ANOVA, df = 3, 44; *F* = 5.4, *P* < 0.003) and worm longevity (ANOVA, df = 3, 236; *F* = 733, *P* < 0.0001). Worms deposited more eggs in DME/F-12 supplemented with either chicken or horse serum than they did in DME/F-12 alone (Figure 3) and lived longer in DME/F-12 supplemented with any of the 3 sera tested than in DME/F-12 alone (Figure 4). Although there was no difference between chicken and horse serum with respect to egg deposition, we observed that a precipitate formed in chicken serum-supplemented medium after a few days of culture.

**Figure 5:**
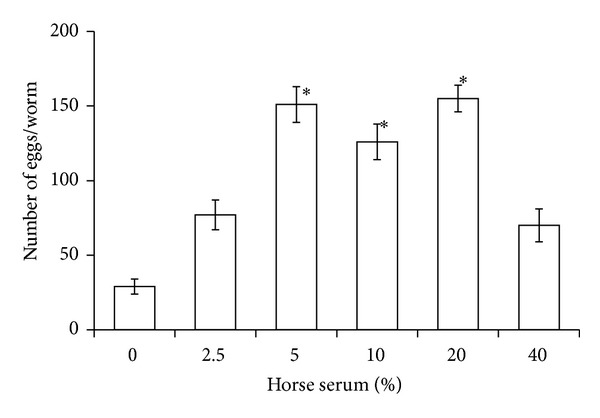
Number of eggs deposited in culture after 10 days at 42 C by* Gynaecotyla adunca* in DME/F-12 supplemented with different concentrations of horse serum. The gas phase was air. Asterisk indicates significantly different from serum-free cultures but not from each other. Each value is a mean ± 1 SE.

Egg deposition was significantly affected by the concentration of horse serum added to DME/F-12 medium (ANOVA, df = 5, 114; *F* = 26.5; *P* < 0.0001). Worms deposited more eggs in medium supplemented with 5, 10, or 20% horse serum than in serum-free DME/F-12. There was no difference between these 3 horse serum concentrations with respect to the number of eggs deposited (Figure 5) or the percentage of normal-shaped, embryonated eggs (mean = 62% ± 5 SE). In both of the serum studies, the percentage of worms with eggs in utero on Day 3 of culture was the lowest when worms were grown in serum-free DME/F-12 (*G*-test, *P* ≤ 0.02 in both cases, data not shown). None of the snails exposed to eggs secreted by* G. adunca* in culture produced cercariae.

## 4. Discussion

To simulate the definitive host environment encountered by adult digeneans, investigators have cultured excysting worms in buffered physiological salt solutions such as HBSS [12] or chemically defined, buffered media that contain various amino acids, carbohydrates, and vitamins. These more complex media include formulations of NCTC medium [12, 20], Eagle's Minimum Essential medium [20], and RPMI-1640 [13, 16, 17]. Though adults of* M. turgidus* are successfully cultured in RPMI-1640, we found that* G. adunca* did poorly in this medium, living less than 4 days and producing few eggs. Consequently, we chose to examine the growth and longevity of the* G. adunca* in DME/F-12. This is an enriched medium, made up of equal parts of Dulbecco's Modified Eagle medium and Ham's F-12 medium, which supports the growth of a broad range of cell types in the presence or absence of serum [21]. We observed that worms grown in DME/F-12 lived longer and deposited more eggs than those grown in RPMI-1640. Consequently, DME/F-12 was used in the subsequent experiments.

Media used for the culture of digeneans are often supplemented with additional nutrients such as host tissue extract [15], blood cells or yeast extract [12, 13], hen's egg yolk [20], or serum [5, 10, 12, 16, 17]. Adults of* G. adunca* grown in DME/F-12 containing chicken or horse serum lived longer and produced more eggs than worms grown in DME/F-12 alone. Newborn calf serum supplementation resulted in increased worm longevity but not egg production. Chicken serum is a common serum supplement for the culture of worms that have avian definitive hosts [3] but we find its use problematical because, at least at the serum concentrations and incubation temperatures we used, a flocculent precipitate, possibly fibrin, formed in the cultures. We tested chicken serum obtained from 2 sources in both RPMI-1640 and DME/F-12 and observed the precipitate in both cases (not shown). Egg production by* G. adunca* worms cultured in horse serum was comparable to that of worms in chicken serum and because the chicken serum precipitate increased the difficulty of counting eggs, horse serum was used in subsequent experiments. Based on the results of egg count studies of worms grown in different concentrations of horse serum-supplemented medium, the optimal concentration of horse serum ranged from 5 to 20%.

Hunter and Vernberg [19] exposed 11 wild-caught* I. obsoleta* snails to eggs from* G. adunca* adults previously maintained in dilute seawater. They reportedly observed mother sporocysts embedded in the gut wall of 3 snails but not the appearance of cercariae. The authors do not indicate if uninfected control snails were included in the study and because of the small sample size, it is difficult to assess the reproducibility of their results. Though we successfully infected hydrobiid snails with eggs from another microphallid,* M. turgidus* [16, 17], grown in conditions similar to those used above for* G. adunca*, our preliminary attempt to infect* I. obsoleta* snails with eggs of in vitro cultured* G. adunca* worms did not succeed. We observed that, like* M. turgidus* [17], excysting* G. adunca* worms are fertilized after overnight incubation with conspecifics in a conical bottom tube. The worms then deposit normal-shaped, embryonated eggs when cultured as described. However, the percentage of worms fertilized and the numbers of embryonated eggs deposited in culture are lower than observed for* M. turgidus* [16, 17]. The percentage of* M. turgidus* worms fertilized in our culture system is typically 2-3 times higher than observed for* G. adunca*. The lower number of fertilized worms is likely responsible for the lower numbers of normal, embryonated eggs deposited by* G. adunca*. Consequently, we are now working to determine the conditions needed to increase the number of worms fertilized. However, the low egg numbers do not explain why none of the snails exposed to eggs from culture became infected. This was possibly due to a combination of factors we are now examining that include egg incubation time, size of infective dose, and exposure technique.

## 5. Conclusion

Culture techniques for digeneans are needed to aid in the design of new anthelmintic drugs and vaccines and to facilitate studies concerned with the biology of the parasites. When cultured in air at 42 C in horse serum-supplemented DME/F-12 medium, excysting metacercariae of the microphallid* G. adunca* readily develop into mature adults that survive for up to 2 weeks and deposit embryonated eggs. These findings are an important initial step in learning how to maintain this and other related digeneans in culture. Further investigation is required to maximize in vitro fertilization of the worms and to determine if deposited eggs are infectious to snail intermediate hosts.
